# Intracytoplasmic morphologically selected sperm injection (IMSI): a critical and evidence-based review

**DOI:** 10.1186/2051-4190-23-10

**Published:** 2013-11-08

**Authors:** Anick De Vos, Nikolaos P Polyzos, Greta Verheyen, Herman Tournaye

**Affiliations:** Centre for Reproductive Medicine, Universitair Ziekenhuis Brussel, Laarbeeklaan 101, B-1090 Brussels, Belgium

**Keywords:** IMSI, High-magnification microscopy, MSOME, Sperm morphology, Sperm nuclear morphology, Sperm nuclear vacuoles, DNA fragmentation, Sperm chromatin, Sperm aneuploidy, Acrosome reaction

## Abstract

Introduced in 2001, intracytoplasmic morphologically selected sperm injection (IMSI) represents a more sophisticated way of ICSI whereby, prior to injection, the spermatozoon is selected at higher magnification. Doing so, the spermatozoon can be evaluated for fine integrity of its nucleus and the injection of a normal spermatozoon with a vacuole-free head can be assured.

Additional research is needed to unravel the underlying mechanisms responsible for the presence of vacuoles in sperm heads. Associations with acrosome status, chromatin condensation, DNA fragmentation and sperm aneuploidy have been documented, however, controversy on their nature exists. Spermatozoon shape and large vacuoles are detected and deselected in conventional ICSI as well. However, the detection of subtle small vacuoles depends on the resolving power of the optical system and may impact oocyte fertilization, embryo development and implantation.

Several comparative studies have indicated that the use of high-magnification sperm selection was associated with both higher pregnancy and delivery rates, whereas also lower miscarriage rates were observed. However, still to date randomized, well-powered studies to confirm these findings are scarce and show conflicting results. Hence, the most relevant indications for IMSI still remain to be determined. Two groups of patients have been put forward i.e. severe male-factor infertility patients and patients with a history of repeated ICSI failures. However, for both groups limited to no proof of any benefit does exist.

IMSI is a time-consuming procedure at the expense of oocyte ageing. The lack of proof and understanding of its benefit does not justify its routine clinical application at present.

## Introduction

Since its introduction in 1992 [1], ICSI is now worldwide used to alleviate male-factor infertility. Whenever possible, ICSI is performed using morphologically well-shaped spermatozoa selected within the limits of the conventional ICSI inverted microscope magnification of x400. However, it became evident that the morphology of the individual spermatozoon used for microinjection into the oocyte is associated to both fertilization and pregnancy outcome [2]. In 2001, Bartoov and colleagues introduced the motile-sperm organelle morphology examination (MSOME). At high magnification the fine nuclear morphology of motile spermatozoa was examined in real time [3]. For this purpose, the inverted light microscope is equipped with high-power differential interference contrast (DIC) optics, resulting in an optical magnification x1500. Further enhancement by digital imaging allows achieving a total magnification of up to x6600. This magnification allows to identify a spermatozoon with a normal nucleus, defined by an oval shape with a smooth configuration and a normal nuclear content (with less than 4% of the nucleus occupied by vacuoles) [3]. Initially, MSOME assessed six sperm organelles (acrosome, postacrosomal lamina, nucleus, neck, tail and mitochondria). However, among the six organelles, the sperm nucleus appeared to be the most important in influencing ICSI outcome [4]. Several publications, mainly from the same group, reported that the selection of spermatozoa with normal nuclear shapes at high magnification was associated with higher pregnancy rates in couples with a history of repeated conventional ICSI failures [5–8].

Additional to nuclear normalcy in terms of shape and size, the impact of nuclear vacuoles in the head of spermatozoa on pregnancy outcome was analysed too [9], showing that microinjection of vacuolated sperm reduced the pregnancy rate and was associated with a higher risk for early abortion. Vacuoles may appear in various numbers and sizes. Small and large vacuoles are well defined presenting a borderline diameter of 0.78 ± 0.18 μm from the front view or larger by 1 SD in length or width [9]. A spermatozoon with a normal nuclear content is defined as having less than 4% of the nucleus occupied by vacuoles. Based on this criterion, Vanderzwalmen et al. [10] established a well-adopted grading system, distinguishing four groups of spermatozoa according to the presence or size of vacuoles [10]. Blastocyst formation was clearly affected when spermatozoa with more than two small vacuoles or at least one large vacuole, with or without additional abnormal head shapes or other abnormalities, were used for microinjection. However, the presence of maximum two small vacuoles in the sperm head did not influence blastocyst development.

The strong need for randomized controlled trials, in order to confirm or refute the initial promising data obtained with IMSI, has so far resulted in a limited number of four studies [11–14]. Based on these studies, the most relevant indications for IMSI are still unclear. It would be clinically relevant to describe the prevalence of vacuoles within semen samples of a given ICSI population and to know their specific impact on oocyte fertilization, embryo development and implantation. Efforts have been made to determine the origin of these vacuoles and to better describe their structure and their location within the sperm head. The presence of large vacuoles in the sperm head has been associated with acrosome status, chromatin condensation, DNA fragmentation and sperm aneuploidy but these reports remain contradictory. The nature and the impact of small vacuoles is less understood. IMSI seems to be a time-consuming procedure, depending on the degree of sperm morphology impairment and the number of oocytes to be injected. The cut-off for the fine morphology of the individual spermatozoon to be selected or deselected by the procedure remains unclear.

### Prevalence of nuclear vacuoles

Vacuoles in human sperm cells appear in various numbers and sizes, both in abnormal-shaped spermatozoa as well as in normal-shaped spermatozoa. About the prevalence of vacuoles within a given sperm sample and within a given ART population, some controversy exists. Earlier studies on IMSI did not report these frequencies, with one exception reporting 33-35% spermatozoa with a vacuolated nucleus [13]. We observed a similar frequency (27.5% grade II and III spermatozoa) within an unselected ART population [15]. However, the majority of spermatozoa showed an amorphous head shape (54.4%). These are easily recognizable and deselected in conventional ICSI as well. The prevalence of normal spermatozoa without any vacuoles was 18% (observation at x1500 without immersion oil, as practical consideration for consecutive microinjection). In contrast, other publications reported lower percentages of normal spermatozoa without vacuoles (1.5-1.8%) [16–18]. The latter observations were made using immersion oil and thus yielding a higher resolution. The total calculated magnification used in these studies was 8400x (total magnification: objective magnification = 100x, magnification selector = 1.0x, video coupler magnification = 1.0x, calculated video magnification = 84.50x).

When selecting spermatozoa using conventional 400x ICSI Wilding et al. [14] reported that 12.1% of them showed multiple vacuoles, while 20.8% showed vacuoles over 4% of the area when assessed at high magnification under immersion oil. Thus, about one in three spermatozoa selected with ICSI would have been deselected by IMSI. Vanderzwalmen et al. [10] showed a similar lower success rate with ICSI in capturing grade I and II spermatozoa as compared to IMSI selection.

The prevalence of vacuoles should be estimated in normal-shaped spermatozoa. Vacuoles were observed in >90% of normally shaped spermatozoa from patient (n = 17) and donor (n = 3) ejaculates [19]. Normally shaped sperm cells without vacuoles or with large vacuoles were very rare in both patient (2.6 and 4.6%, respectively) and fertile donor samples (0.0 and 4.2%, respectively) [19]. In contrast, the prevalence of small vacuoles found in normally shaped spermatozoa was extremely high (92.8% in patients and 95.8% in fertile donors) [19]. Similar high frequencies of vacuoles of various sizes in ejaculated sperm samples were observed both by Tanaka et al. (97.4%) [20] and by Perdrix et al. (98-99%) [21]. Vacuoles were mainly located in the tip or middle area of the sperm heads [19]. A similar anterior and median location was observed for large vacuoles in teratozoospermic patients [22]. A higher prevalence of large vacuoles (38%) was observed in this specific patient population [22].

Regarding their significance in terms of oocyte fertilization and further embryo development, a sibling oocyte study showed a lower fertilization rate with grade II spermatozoa (normal-shaped, containing no more than 2 small vacuoles, 67.4%) than with grade I spermatozoa (normal-shaped, without vacuoles, 78.9%) [15]. However, this study did not show any difference in total blastocyst formation once the oocyte was fertilized. Beyond blastocyst formation, the implantation rate per embryo was not affected when the embryos were derived from grade II spermatozoa (four implantations with fetal heartbeat were obtained transferring eight embryos in seven transfer cycles) as compared to an implantation rate of 27.2% with embryos derived from grade I spermatozoa (147 embryos transferred in 118 transfer cycles) [15]. If the present finding could be substantiated, this would argue against deselecting these spermatozoa with one or few small vacuoles and thus advanced selection at higher magnification using more sophisticated equipment would be unnecessary.

### Nature of the so-called nuclear vacuoles

Despite the increasing interest in the use of IMSI as an alternative to conventional ICSI, it is poorly understood how the presence of vacuoles (single, multiple, large or small) in the sperm head (or their absence) may affect the clinical outcome.

The origin of large vacuoles in spermatozoa (in contrast to small ones) has been characterized recently [23]. In all vacuolated spermatozoa, the acrosome was intact, the plasma membrane was sunken but intact, the large vacuole was identified as an abnormal, ‘thumbprint’-like nuclear concavity covered by acrosomal and plasmic membranes [23]. The exclusively nuclear character of large vacuoles has been confirmed by others [21, 22], although an acrosomal origin has also been suggested [24]. Sperm vacuoles have been linked to a non-reacted acrosomal status of the spermatozoon [24, 25]. According to the authors, MSOME selection would, by elimination of vacuolated spermatozoa, favour the injection of acrosome-reacted spermatozoa.

Boitrelle and colleagues [23] found that the rate of non-condensed chromatin was higher for vacuolated spermatozoa. However, no significant difference in terms of DNA fragmentation or aneuploidy was observed between vacuolated and vacuole-free spermatozoa. Several publications agree that large nuclear vacuoles are related to chromatin condensation defects [22, 26–28] (Table 1). Regarding DNA fragmentation, however, some authors reported increased DNA fragmentation in vacuolated spermatozoa [14, 18, 29, 30], whereas this has not been confirmed by others [19, 22, 23, 28] (Table 1). Possible explanations for these contradictory findings may be related to the methodology: assay specificity [19, 30], inclusion of dead spermatozoa in unselected samples [22] and subjective fluorescence microscope analysis of TUNEL slides [22]. Additionally, patient populations studied showed different sperm characteristics, sperm types (normal and abnormal spermatozoa with large vacuoles) and vacuole sizes (ranging from 15% of the head’s cross-sectional area to over 50%) [19, 22, 30].

**Table 1:** **Studies on the relationship between the presence of large sperm vacuoles and chromatin condensation, DNA fragmentation and aneuploidy**

References	Patients	Sperm cells	Vacuoles	Altered/abnormal chromatin packaging	DNA fragmentation	Aneuploidy
Boitrelle et al., [23]	15	450	≥ 25% head area	+	=	=
Perdrix et al., [22]	20		>13% head area	+	Higher in native spermatozoa	+
Garolla et al., [26]	20	200	LNV	+	NA	+
Franco et al., [27]	66	1351	≥ 50% head area	+	NA	NA
Cassuto et al., [28]	26	10400	score 0^a^	+	=	NA
Wilding et al., [14]	8	860	≥ 4% head area	NA	+	NA
Oliveira et al., [18]	538	200/patient	5- > 50% head area	NA	+	NA
Franco et al., [29]	30	382	≥ 50% head area	NA	+	NA
Hammoud et al., [30]	8	1775	>4% head area	NA	+	NA
Watanabe et al., [19]	20	227	>1.5 μm	NA	=	NA
		33	>1.5 μm	NA	NA	=

LNV, large nuclear vacuole; NA, not assessed.

^a^score 0, according to the Cassuto-Barak classification, showing an abnormal head with a large diameter vacuole and an abnormal base. For DNA fragmentation and aneuploidy: =, equal between vacuolated and non-vacuolated spermatozoa ; +, increased in vacuolated spermatozoa.

Whereas correlations between aneuploidy rates and the presence of vacuoles in the sperm head have been reported [22, 26], this finding was not confirmed by Boitrelle and colleagues [23]. Neither did Watanabe et al. [19] observe an increased incidence of structural chromosome aberrations in sperm cells exhibiting large vacuoles (Table 1). When evaluating the incidence of aneuploidy in embryos derived from ICSI and IMSI treatment [31] however, the incidence of sex chromosomal aneuploidy was higher in ICSI embryos than in IMSI embryos (23.5% vs. 15.0%). The autosomal aneuploidy rate was not affected by the sperm selection. The incidence of chaotic embryos was also significantly higher with the conventional ICSI procedure. These observations need to be substantiated, distinguishing between sperm cell and oocyte contribution to the chromosomal content of the embryo.

### Practical issues and safety considerations

MSOME requires the use of glass-bottomed dishes. Sperm suspensions are transferred into a droplet containing PVP covered with paraffin or mineral oil. Oocytes to be injected are contained in separate microdroplets in the same dish. Most researchers have used an oil objective lens in combination with immersion oil in order to achieve the highest resolution. However, others have used a dry objective lens without immersion oil [15, 20, 23]. It has been recommended to perform the sperm examination at room temperature [32], because prolonged manipulation at 37°C resulted in an increased incidence of spermatozoa with vacuolated nuclei.

MSOME has been described in conjunction with hyaluronan [12, 33], a major component of the cumulus oophorus matrix that may play a critical role in the selection of functionally competent spermatozoa [34]. Hyaluronan-bound spermatozoa show lower rates of DNA fragmentation [35], a normal nucleus [35] and display a reduced frequency of chromosomal aneuploidies [34]. Additionally, they have completed the spermiogenic process [34].

Several publications have indicated that MSOME sperm selection is rather time-consuming [6, 8, 10, 11, 20, 26]. The average duration of the process was about 2.5 hours (range 1.5-5.0) as reported by Berkovitz et al. [6] for an average of 10 oocytes to be injected. Hazout et al. [8] also reported 30 minutes to 2 hours, depending on the degree of impairment of sperm morphology. Vanderzwalmen et al. [10] used 2 to 15 minutes to select the best spermatozoon, noting that it is difficult to decide when to stop the search for a normal spermatozoon (15 minutes or longer) and divert to second-best spermatozoa with the least number of vacuoles and/or other abnormalities. Similarly, about 30 minutes per sperm cell was reported by Garolla et al. [26]. Finding normal-looking spermatozoa took a minimum of 60 minutes and up to 210 minutes for only three oocytes to be injected according to the Italian law at that time [11]. Facing the high frequency of spermatozoa with vacuoles, it is a very difficult and sometimes impossible process finding those without vacuoles [20]. Despite a lower fertilization rate, grade II spermatozoa might perform similar to grade I spermatozoa in terms of blastocyst formation and implantation [15]. The selection process could thus be shortened with a lower cut-off level for nuclear normalcy, given that spermatozoa with a few small vacuoles perform equally well as compared to vacuole-free spermatozoa in supporting embryo development and implantation. Sperm injection should not be delayed in order to avoid oocyte ageing.

In contrast, other studies have performed the selection of spermatozoa in shorter periods of time [36]. These are mainly studies evaluating whole semen samples without consecutive oocyte microinjection for clinical purposes. Two hundred spermatozoa per sample are then evaluated, lasting 30–60 minutes per sample [36]. However, if only 1.2 ± 2.0% (range 0-15%) represent normal forms, on average only two spermatozoa suitable for oocyte microinjection would have been recovered (range 0-30 spermatozoa) during that time period, comparable to Garolla et al. [26].

Most recently, a higher frequency of congenital abnormalities and a lower birth weight were reported following IMSI compared with ICSI [37] however, these differences were not statistically significant. Moreover, these findings have not been confirmed by others. Indeed, long-term follow up for anomalies in the offspring are currently lacking [38] and should be conducted in order to provide assurance regarding the use of advanced sperm selection. Additionally, it was reported that IMSI influenced the sex ratio of the offspring [31, 39], however, this finding has neither been confirmed by others [40] based on six years of experience with IMSI in three centers.

### IMSI and embryo development

Contradictory reports exist on whether IMSI improves embryo development or not. Some of the earlier comparative studies reported improved embryo development with IMSI [5, 7] while others reported equal embryo development with IMSI and ICSI [8, 9]. The clinical trials on IMSI that do report on embryo development remain contradictory. Two of them observed improved embryo development with IMSI [16, 41], but others did not confirm these findings [12, 13].

Furthermore, very few studies have specifically evaluated embryo development when IMSI was applied [10, 15, 33, 42, 43]. Most of these studies agree on comparable embryo development between IMSI and ICSI on day 2 [42] and on day 3 [10, 15, 33, 43]. For day 5 blastocyst formation after injecting vacuolated spermatozoa, data remain limited (25 patients, 143 zygotes) [10], showing that blastocyst formation was negatively influenced by the use of grade III and grade IV vacuolated spermatozoa as compared to the use of grade I (no vacuoles) and grade II (≤ two small vacuoles) spermatozoa (respectively 5% and 0% as compared to 56% and 61%). These data have been confirmed by Knez et al. [12], showing decreasing blastocyst formation in 30 patients according to the grade of spermatozoa injected.

In our sibling oocyte study [15], no difference in blastocyst formation was observed between IMSI and ICSI. In the IMSI subgroup, almost all oocytes were injected with grade I and grade II spermatozoa (90.4% and 8.5% respectively), whereas the obligatory use of grade III and IV spermatozoa was restricted to only 1.1% of the oocytes (or only five patients in a cohort of 340 patients). Standard ICSI was performed on the other half of the oocytes and blastocyst formation rate was similar. This can only be explained by the fact that both grade III and grade IV spermatozoa can easily be recognised at 400x magnification, and will not be selected for standard ICSI, unless no other spermatozoa are available.

### IMSI and clinical outcome

In the earlier days of IMSI, its possible advantage in terms of pregnancy has mainly been shown in case–control studies, mainly in patients with repeated conventional ICSI failures [5–9], reviewed by Nadalina et al. [44]. None of these studies observed a difference in oocyte fertilization rate between ICSI and IMSI. Whether embryo development was improved by performing IMSI remained unclear [5, 7–9]. Yet, significantly higher implantation and pregnancy rates were reported after IMSI, as well as significantly lower abortion rates.

But randomized controlled trials are still scarce today [11–14], are sometimes underpowered and have been conducted only in cases of male-factor infertility [11, 12] or in unselected infertile populations [13, 14]. None of the studies showed a difference in fertilization rate between ICSI and IMSI treatment. The largest study on male infertility patients reported a higher clinical pregnancy rate with IMSI compared to ICSI, whereas miscarriage rates were not different between both procedures [11]. A maximum of three oocytes per patient was injected, according to the Italian law at that time. Knez and colleagues [12] confirmed an improved clinical pregnancy rate using teratozoospermic samples, with an average of 10–11 oocytes injected per patient. Similar findings were obtained in a more recent, prospective non-randomized observational study [45]. Significantly higher implantation and clinical pregnancy rates were obtained with IMSI in a patient population with severe teratozoospermia [45]. In contrast, the authors reported no differences between ICSI and IMSI in patients with at least two previously failed conventional ICSI attempts [45]. So far, there are no randomized studies available in groups of repeated ICSI failure patients. However, Oliveira and colleagues [16] reported no significant differences between ICSI and IMSI with regard to fertilization, implantation and pregnancy rates in a comparative study. Miscarriage rates were also similar for ICSI and IMSI.

In unselected infertile populations, IMSI did not significantly improve the clinical outcome as compared to ICSI [13]. This was confirmed in a more recent pilot study [41], however, contradicted by Wilding et al. [14].

**Figure 1:**
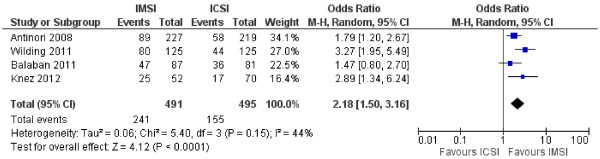
**Meta-analysis comparing IMSI and ICSI for clinical pregnancy rate, expressed as odds ratios (OR) with 95% confidence intervals (CI).** Four randomized studies were included. Two studies included male-factor infertility patients (Antinori et al. [11]; Knez et al. [12]), while two studies dealt with unselected patient populations (Wilding et al. [14]; Balaban et al. [13]).

Pooled analysis of data from the four existing randomized studies [11–14] was performed in order to check the cumulative odds ratio for clinical pregnancy rate after treatment with IMSI versus ICSI (Figure 1). By using the random effects model, couples undergoing IMSI demonstrated a higher likelihood for clinical pregnancy OR 95%CI 2.18 (1.50-3.16), p < 0.0001 (Figure 1). Although cumulative analysis demonstrated a benefit, results should be interpreted with caution, firstly due to the small number of patients and trials and secondly due to the clinical heterogeneity of the populations included in these trials.

### IMSI has no place in routine practice

Based on the above clinical findings, the IMSI procedure might represent a valuable option for patients with severe teratozoospermia [11, 12, 45]. However, if no normal spermatozoa can be found using MSOME, the only alternative is to choose morphologically second-best ones. It should be kept in mind that live births have been obtained with the use of morphologically amorphous spermatozoa, although to a lesser extent than with morphologically normal ones [2].

For unselected infertile patients contradictory results [13, 14, 41] argue against a widespread adoption of this technique into routine laboratory practice. Sperm selection by conventional ICSI seems sufficient for an unselected population [46], as evidenced by similar pregnancy and delivery rates for ICSI and IMSI in the very first ART cycle of a couple (retrospective cohort study).

In the second cycle subsequent to a failed ICSI, however, the same cohort study reported significantly higher pregnancy and delivery rates for patients who shifted to the IMSI technique compared to patients who had a second standard ICSI cycle [46]. Therefore, the authors concluded that the IMSI procedure is a good option for couples with a first unsuccessful ICSI cycle [46]. Again, so far, there are no randomized trials to confirm this strategy. Moreover, under non-randomized conditions, IMSI did not improve pregnancy rates in patients with repeated ICSI failures in the absence of a severe male factor [16, 45]. The poorest success rates were obtained in couples failing their first IMSI cycle and choosing to carry on with this method [46]. Thus, repeated IMSI cycles seem to be of no use.

IMSI has been used in a case of globozoospermia allowing the selection of spermatozoa with a small bud of acrosome [47]. A successful pregnancy and healthy childbirth has been obtained, even without assisted oocyte activation. A higher magnification may aid the selection of spermatozoa with the slightest presence of acrosomal material, however, the additional value over conventional ICSI in these specific cases of globozoospermia has not been established.

## Conclusions

Obviously, every single good-quality oocyte deserves the best spermatozoon available in the sperm sample to be used for microinjection, in order to obtain the highest probability of developing a high quality embryo that implants.

It needs to be defined what is absolutely needed in terms of nuclear normalcy presenting with no single vacuole in the sperm head on the one hand and what is at least as good or good enough to be used as second-best spermatozoon on the other hand without compromising oocyte fertilization, embryo development and implantation potential. In other words, how much time, what skills and sophisticated expensive equipment should be invested to achieve this goal? Large vacuoles, although less frequent, are well-characterized so far, but these are recognized in conventional ICSI as well. Instead, the nature of the more abundant small vacuoles is less understood, as well as their impact on oocyte fertilization, embryo development and implantation.

As the technique seems not effective for any unselected ART patient, relevant indications for the use of IMSI need to be defined. For severe male factor patients, evidence suggests a higher clinical pregnancy rate with IMSI. The benefit for repeated ICSI failure patients, however, remains unproven.

## Abbreviations

ART: Artificial reproductive technology
DIC: Differential interference contrast
DNA: Deoxyribonucleic acid
ICSI: Intracytoplasmic sperm injection
IMSI: Intracytoplasmic morphologically selected sperm injection
MSOME: Motile sperm organelle morphology examination
PVP: Polyvinylpyrrolidon
SD: Standard deviation
TUNEL: Terminal deoxynucleotidyl transferase dUTP nick end labeling.
